# Coupling fermentation of glutamic acid and γ-polyglutamic acid and preparation of poly(amino acid) superabsorbent polymers

**DOI:** 10.1186/s12896-023-00819-0

**Published:** 2023-10-31

**Authors:** Zhao Jianbo, Wei Jun, Wang Xuanlin, Cao Hui

**Affiliations:** 1 Engineering Laboratory of Chemical Resources Utilization in South Xinjiang of Xinjiang Production and Construction Corps, College of Chemistry and Chemical Engineering, Tarim University, No.1487 Tarim East Avenue, Alar, Xinjiang, 843300 PR China; 2 Beijing Key Laboratory of Biochemical Engineering, Beijing University of Chemical Technology, No.15 Beisanhuan East Road, Chaoyang District, Beijing, 100029 PR China

**Keywords:** γ-polyglutamic acid, Polyaspartic acid, Coupling fermentation, Superabsorbent polymer

## Abstract

γ-polyglutamic acid (γ-PGA) is a biomarker that can be directly obtained by microbial fermentation. Poly(amino acid) superabsorbent polymers (SAPs) were prepared with purified γ-PGA as raw material and ethylene glycol diglycidyl ether (EGDGE) as a cross-linking agent. However, γ-PGA fermentation broth has a high viscosity, requires complex extraction and separation processes, and entails high energy consumption, resulting in the high cost of poly (amino acid) SAPs. Therefore, the coupling fermentation processes of glutamate polyglutamic acid, the process of using glutamate fermentation broth instead of pure glutamate powder for fermentation, and the process of treating the fermentation broth under conditions of centrifugation, UV irradiation, and high temperature, were studied. The results showed that the yield of γ-PGA after centrifugation decreased by 5%, but it did not affect the synthesis of hydrogels, and the addition of γ-PGA fermentation broth had a significant effect on the performance of γ-PGA-co-PASP SAPs. The proposed method not only helps avoid the separation of complex γ-PGA fermentation broth and reduces the cost, but it also helps improve the performance of the super-absorbent resin, which has great application potential.

## Introduction

Superabsorbent polymers (SAPs) are a type of three-dimensional, cross-linked hydrophilic polymer that can absorb and retain large quantities of pure water or saline physiological fluids [1–3]. Their excellent properties mean that SAPs have been widely used in agriculture, forestry, industry, drug-delivery systems, cartilage tissue engineering, wound healing, and hygienic products [4–8]. Currently, most of the commercially available SAPs are synthetic polyacrylic acid and polyacrylamide or starch-acrylic acid copolymers [9–11]. However, those polymers are poorly biodegradable, and their degradation products are hazardous to the environment [12, 13]. Therefore, environment-friendly, and completely biodegradable SAPs such as starch, cellulose, chitosan, and poly (amino acid) have become the focus of current research [14–16].

As one type of poly(amino acid) compound, γ-polyglutamic acid (γ-PGA) is formed from glutamic acid through the amide bond of the α-amino group and γ-carboxyl group [17]. The preparation of PGA superabsorbent resin is divided into three steps: First, glucose is used as a raw material to ferment with *Corynebacterium glutamicum* followed by separation and purification to obtain glutamate. Second, purified glutamate is fermented with *Bacillus subtilis* to obtain γ-PGA fermentation broth [18]. Third, γ-PGA is extracted and separated from the fermentation broth, and then it is added with ethyl glycol diglycidyl ether (EDGDE), a crosslinking agent, to prepare γ-PGA SAPs [19]. The material exhibited good water absorption and water retention abilities. It also exhibited good biocompatibility and biodegradability and can be used in the fields of biomedicine, environmental protection, and agricultural production [20–22]. Because of the extremely high viscosity of the γ-PGA fermentation broth, complex extraction and separation processes are required to extract γ-PGA macromolecules from the fermentation broth. The process is accompanied by high energy loss, resulting in high costs, which limits the application of the γ-PGA SAPs [23]. In addition, the separation process associated with the glutamate fermentation broth is also accompanied by a large amount of energy. We have previously reported the synthesis of polyaspartic acid (PASP) superabsorbent polymers, a kind of poly(amino acid), using water as the solvent and EGDGE as a cross-linker [24–26].

**Fig. 1:**
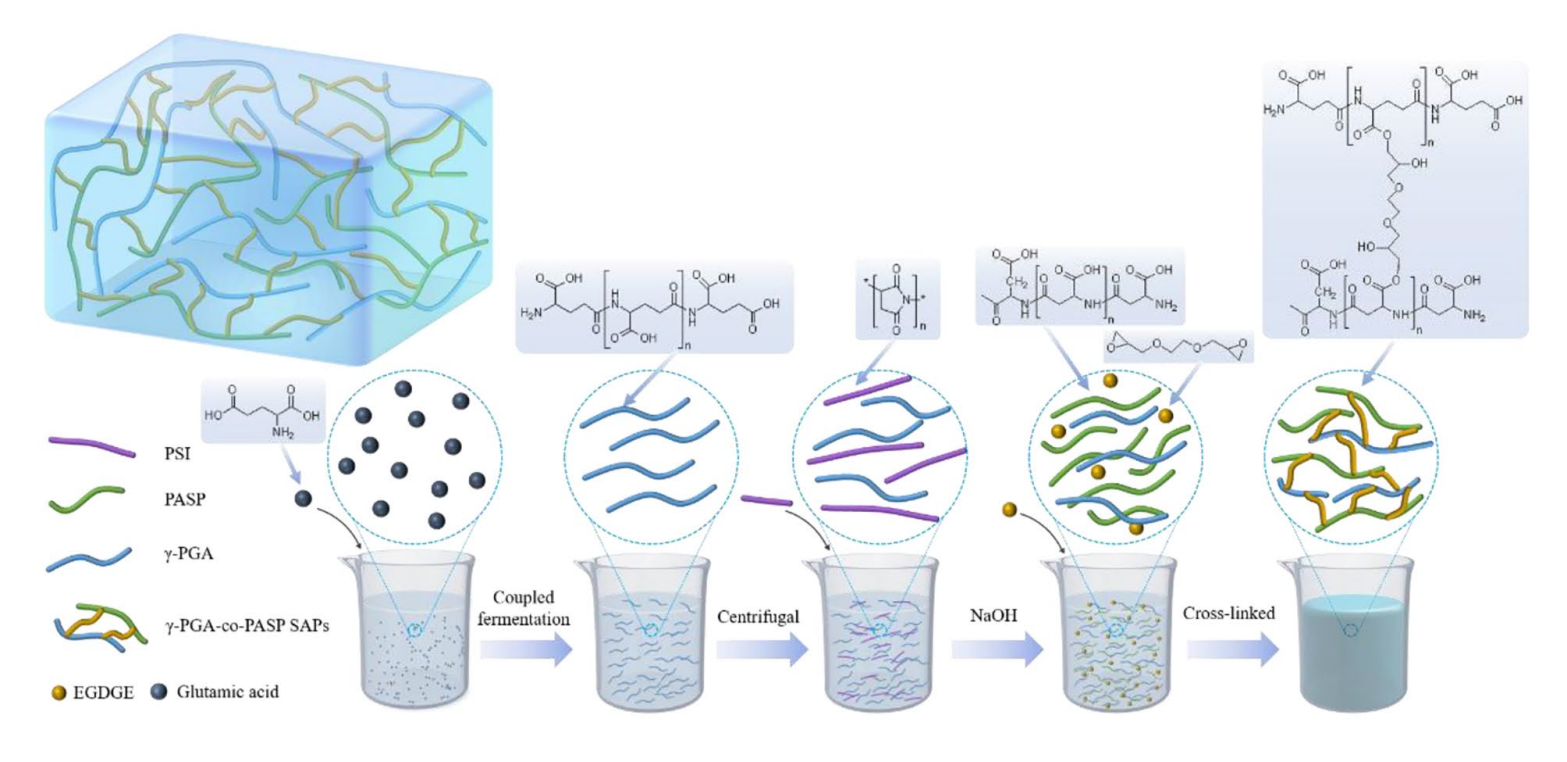
poly(amino acid) SAPs preparation route

Here, we report a preparation method of an environmentally friendly and completely biodegradable copolymer that is composed of γ-polyglutamic acid and polyaspartic acid (γ-PGA-co-PASP) SAPs. Glutamic acid was produced via fermentation with glucose as a raw material, and then γ-PGA was produced via fermentation after centrifugation, ultraviolet irradiation, or high-temperature heating pretreatment of glutamic acid fermentation broth. Finally, untreated γ-PGA fermentation broth was used to replace part of the water, and EGDGE was used as a cross-linking agent to prepare poly(amino acid) SAPs. This method can reduce the expense of separation and purification of glutamic acid and γ-PGA, and limit the process steps, which provides a new strategy for the preparation of poly(amino acid) SAPs. The preparation route of poly(amino acid) SAPs is shown in Fig. 1.

## Materials and methods

### Materials

Polysuccinimide (PSI, *M*_w_ ≈ 100,000) was prepared by Luoyang Kang’en Chemical Company. EGDGE was obtained from Shanghai RuFa Chemical Technology Corporation. Sodium hydroxide, phosphoric acid, and ethanol were of analytical grade and purchased from Beijing Chemical Reagent Company. The water used for the experiments was previously deionized. *Corynebacterium glutamicum* (No. 10,109) and *Bacillus Subtilis* (No. 20,643) were used, and the strain was deposited in the China Centre for Industrial Culture Collection. The glutamic acid fermentation medium contained glucose (80 g/L), Na_2_HPO_4_·12H_2_O (3 g/L), MgSO_4_·7H_2_O (1.8 g/L), KCl (1.7 g/L), methionine (2 g/L), MnSO_4_·H_2_O (2.5 mg/L), FeSO_4_·7H_2_O (2.5 mg/L), Vitamin B_1_ (0.5 mg/L), molasses (1 g/L), corn steep liquor (4 mL/L), and soybean cake meal hydrolysate (20 mL/L). The γ-PGA fermentation medium contained glutamic acid fermentation broth (30 g/L), glucose (20 g/L), citric acid (20 g/L), (NH_4_)_2_SO_4_ (5 g/L), K_2_HPO_4_·3H_2_O (2.5 g/L), MgSO_4_ (0.15 g/L), and MnSO_4_ (0.04 g/L). The pH was controlled to within 7.3 ± 0.1.

### Coupling fermentation of glutamic acid and γ-PGA

The glutamate fermentation broth was pretreated separately with centrifugation, UV irradiation, and high temperature. For pretreatment by centrifugation, 5% (w/V) diatomite was added to the fermentation broth and centrifuged at 8000 rpm at 4 °C for 30 min. For pretreatment by UV irradiation, the fermentation broth was placed on an ultra-clean table for more than 30 min, and the wavelength was 280 nm. For high temperature pretreatment, the fermentation broth was heated in a 50 °C water bath for 45 min. After pretreatment using the three different methods, the pretreated glutamate fermentation broth was added to a polyglutamic acid fermentation medium, and γ-PGA was prepared following the process of coupling fermentation. The medium was autoclaved at 116 °C for 20 min. *Bacillus subtilis* was inoculated at 2%, fermented at 30 ± 0.5 °C for 24 h, and oscillated at 180 rpm in a constant temperature oscillator. The feeding medium containing 500 g/L of glucose was used to maintain a constant glucose concentration of 20 g/L.

### Preparation of poly(amino acid) superabsorbent polymers

The preparation of PASP has been previously reported, which involves dissolving PSI in an alkaline aqueous solution and then crosslinking it with EGDGE, a crosslinking agent [27, 28]. The poly(amino acid) superabsorbent polymers were produced by dispersing 3.0 g of PSI in 3 mL γ-PGA fermentation broth and 15 mL deionized water (total volume of the solution: 18 mL) under conditions of magnetic stirring in a 50-mL beaker. Then, 1.44 g NaOH was added to the beaker. After stirring the mixture for 30 min, the solution pH was adjusted in the range of 4.8–5 using phosphoric acid. The poly(amino acid) hydrogel can be obtained by adding 1.5 g of EGDGE at a reaction temperature of 50 °C (stirring time: 12 h). Finally, the hydrogels were oven-dried at 60 °C to constant mass to yield poly(amino acid) SAPs.

### Analytical methods

The glutamic acid and glucose concentrations in the broth were measured enzymatically using a biosensor (SBA-40 C, China). Dry cell weight (DCW) was determined by centrifugation for 20 min at 4 °C to precipitate cell suspension. The samples were then washed with distilled water and dried at 80 °C to achieve constant weight. The concentration of γ-PGA was measured following the gel permeation chromatography (GPC) method. The amount of γ-PGA was calculated from the peak area measured using the GPC technique using NaCl aqueous solution–acetonitrile (50 mM; 4:1, v/v) as the mobile phase at a flow rate of 0.6 mL/min. Purified PGA was used as the standard.

### Determination of the properties of the poly(amino acid) SAPs

#### Swelling ratio

The swelling behavior of the poly(amino acid) SAPs was studied following the tea-bag method [27, 28]. First, the samples were soaked in excessive distilled water for seven days to remove inorganic ions and small molecules from bacteria, following which the samples were freeze-dried. The dry sample (0.1 g) was placed in a tea bag (300-mesh, nylon, 40 mm in length, and 50 mm in height), and the bag was fully immersed in excess deionized water at room temperature. At a fixed interval, the tea bag was removed and hung in the air for 5 min to remove moisture. The swelling ratio (SR) was calculated using Eq. 1 as follows:

1 $$SR=(W_{t} - W_{0} - W_{n})/W_{0}$$

where *W*_t_, *W*_0_, and *W*_n_ are the weights of the teabag, including the hydrated polymer, the dried sample, and the wet nylon net, respectively.

#### SEM observation

The surface morphologies of poly(amino acid) SAPs were observed by a scanning electron microscope (SEM) (Hitachi S-4700, Japan). The freeze-drying method was used to observe the swollen internal structure of the SAPs. The samples were soaked in excess deionized water for seven days to allow complete swelling, and the samples were stored in a refrigerator and freeze-dried for 24 h at − 45 °C. Subsequently, the freeze-dried sample was removed, and the surface was coated with gold for SEM observation.

#### Thermal properties

Thermogravimetric analysis (TGA) was carried out on a METTLER TGA/DSC instrument (SF/1382) under a nitrogen atmosphere. The samples were placed in an aluminum cup and sealed, and an empty pan was used in the experiment. The samples were heated from room temperature to 800 °C at a rate of 10 °C/min. Nitrogen gas was used to confirm the thermal behavior.

#### The rheological characteristic curve

The storage modulus (*G*´) and loss modulus (*G*´´) of the poly (amino acid) SAPs were measured using an Anton Paar instrument (Physical MCR 301, Germany) equipped with a standard rotor (diameter 20 mm). The moduli were read under the strain sweep mode, the fixed frequency was 1 Hz, the strain was increased from 0.001 to 100%, and the moduli of the SAPs were determined. Subsequently, the moduli were read under the angular frequency sweep mode at a constant shear strain of 0.1%, and the angular frequency was increased from 1 to 100 rad/s.

## Results and discussion

### Coupling fermentation of glutamic acid and γ-PGA

The glutamic acid fermentation broth contained a large amount of *Corynebacterium glutamicum*, and the use of untreated glutamic acid fermentation broth resulted in a competition between *C. glutamicum* and *Bacillus subtilis*, which was not conducive to the synthesis of γ-PGA [29]. Therefore, it was necessary to remove *C. glutamicum* or reduce its activity so that the subsequent synthesis process could proceed smoothly. In the present research, centrifugation, UV irradiation, and heating methods were executed at 50 °C for pre-treatment. Following this, the contents of glutamic acid and DCW produced by *C. glutamicum* in the fermentation broth were determined.

**Fig. 2:**
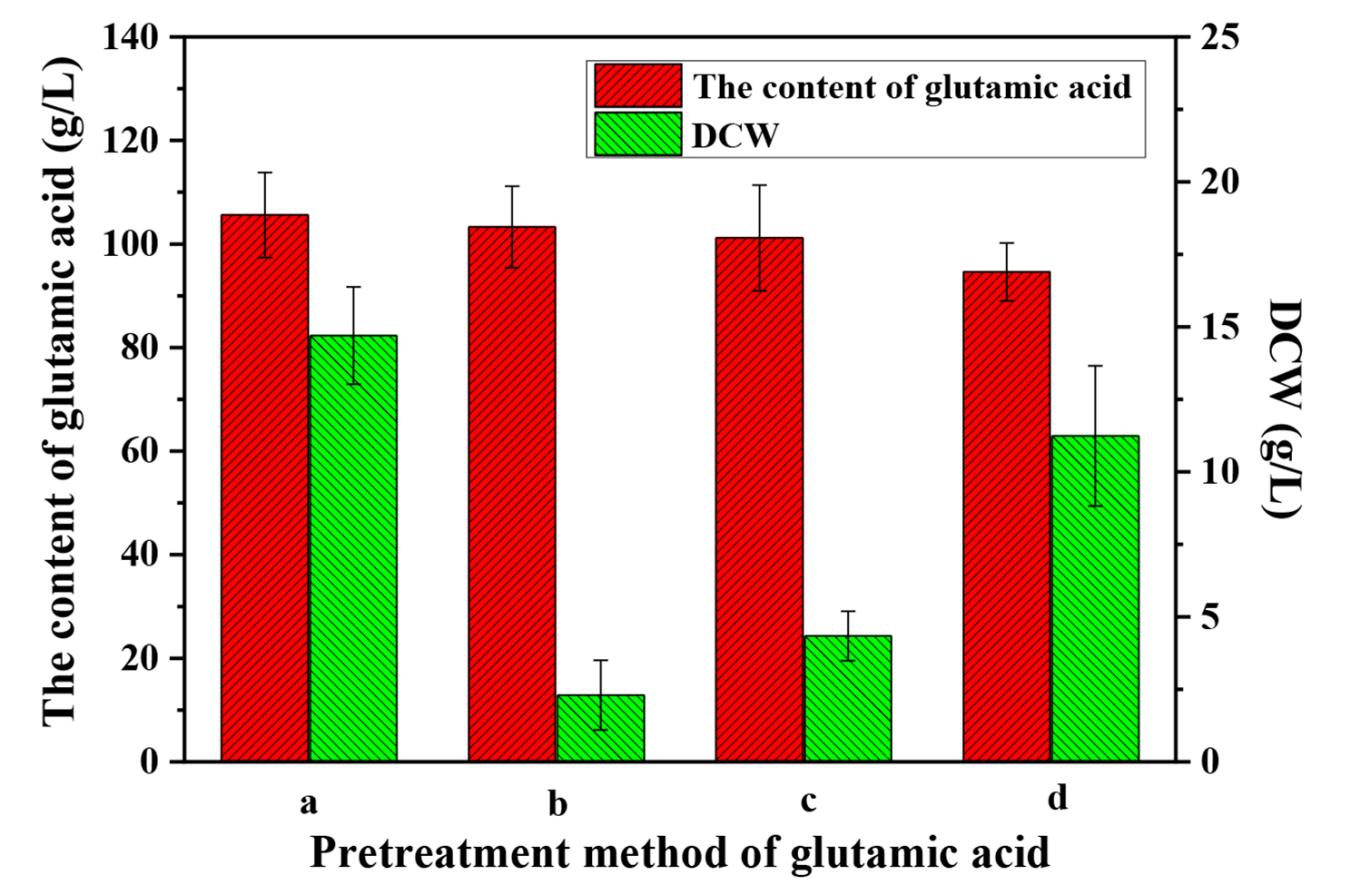
Glutamic acid content and DCW in glutamic acid fermentation broth (a. before pre-treatment; b. pre-treatment by centrifugation; c. pre-treatment by UV irradiation; d. pre-treatment at 50 °C)

Figure 2 shows the content of glutamic acid and DCW in the glutamic acid fermentation broth before and after pre-treatment. It can be seen that the concentration of glutamic acid did not change significantly, and the DCW decreased to 2.3 g/L after centrifugation. Following UV irradiation, the glutamic acid concentration remained unchanged, and the DCW decreased to 4.34 g/L. When heated to 50 °C, the content of glutamic acid decreased to a certain extent, primarily due to the Maillard reaction of glucose and glutamic acid under conditions of heating [30]. The DCW decreased to 11.25 g/L, and the inactivation effect was poor, and this could be attributed to the low-temperature pre-treatment process.

The pre-treated glutamic acid fermentation broth was used instead of glutamic acid, and then *Bacillus subtilis* was added to produce polyglutamic acid. The glutamic acid-fermented polyglutamic acid was used as a control, and the content of γ-PGA, glutamic acid, and DCW produced by *Bacillus subtilis* was determined at a fixed time interval.

**Fig. 3:**
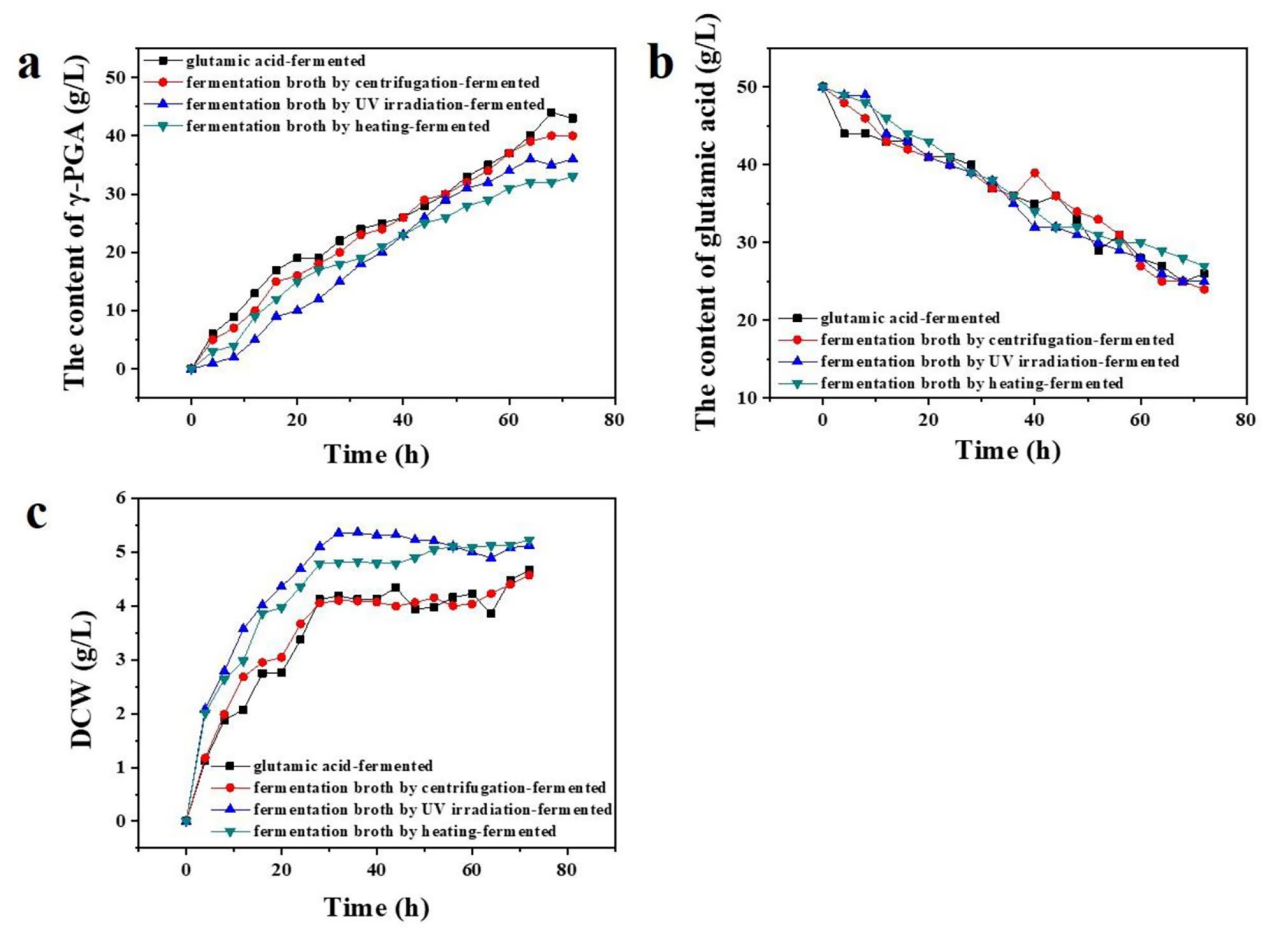
Changes in various indicators during the fermentation process of polyglutamic acid. (**a**) Variation of γ-PGA content with time; (**b**) Variation of glutamic acid content with fermentation time; (**c**) Variation of DCW with fermentation time

The yield of γ-PGA, the content of glutamic acid, and DCW recorded during the fermentation process conducted in the glutamic acid fermentation agreed well with the results obtained when pure glutamic acid was fermented (Fig. 3). Therefore, glutamic acid fermentation broth can be used as a replacement of pure glutamic acid during the production of γ-PGA. Compared with the results obtained from pure glutamic acid at 72 h, the concentration of γ-PGA obtained from the glutamic acid fermentation broth under conditions of centrifugation was 40 g/L, which was lower by 5%, and the DCW value was 4.57 g/L, which was lower by 2%. The γ-PGA produced from the UV-pre-treated glutamic acid fermentation broth was 36 g/L, which was lower by 17%, while DCW increased slightly. The production of γ-PGA from the glutamic acid fermentation broth pre-treated under conditions of heating was 33 g/L, which was lower by 23.3%. The increase in DCW may be due to the presence of *Corynebacterium glutamicum* in the fermentation broth, which promotes fermentation [31]. Moreover, compared to the glutamic acid fermentation broth subjected to conditions of centrifugation, the time required for the synthesis of γ-PGA using the UV irradiation-treated and heating-treated systems was delayed by 4 h. In summary, the pre-treatment of glutamic acid fermentation broth under conditions of centrifugation can avoid a decrease in γ-PGA production caused by competition between the two bacteria. The process can reduce the production cost, which is of practical value.

**Fig. 4:**
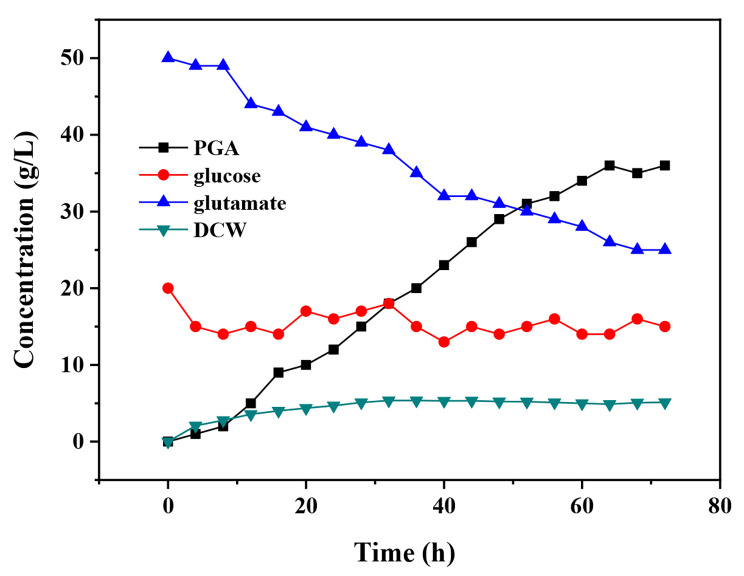
Coupling fermentation time curve of glutamate fermentation broth after centrifugation

Figure 4 demonstrates the change trends of glutamate content, polyglutamic acid content, glucose content, and DCW in the coupling fermentation process after centrifugation of glutamate fermentation broth: as fermentation time increased, the content of glucose in the system changed little, the content of glutamate decreased gradually, and more polyglutamic acid was produced. The centrifuged glutamate fermentation broth could be used to replace the pure glutamate powder without affecting the overall fermentation.

### Water absorption

According to the amount of PSI and the volume of the γ-PGA fermentation broth, a series of γ-PGA-co-PASP SAPs were prepared, and their water absorption was determined.

**Fig. 5:**
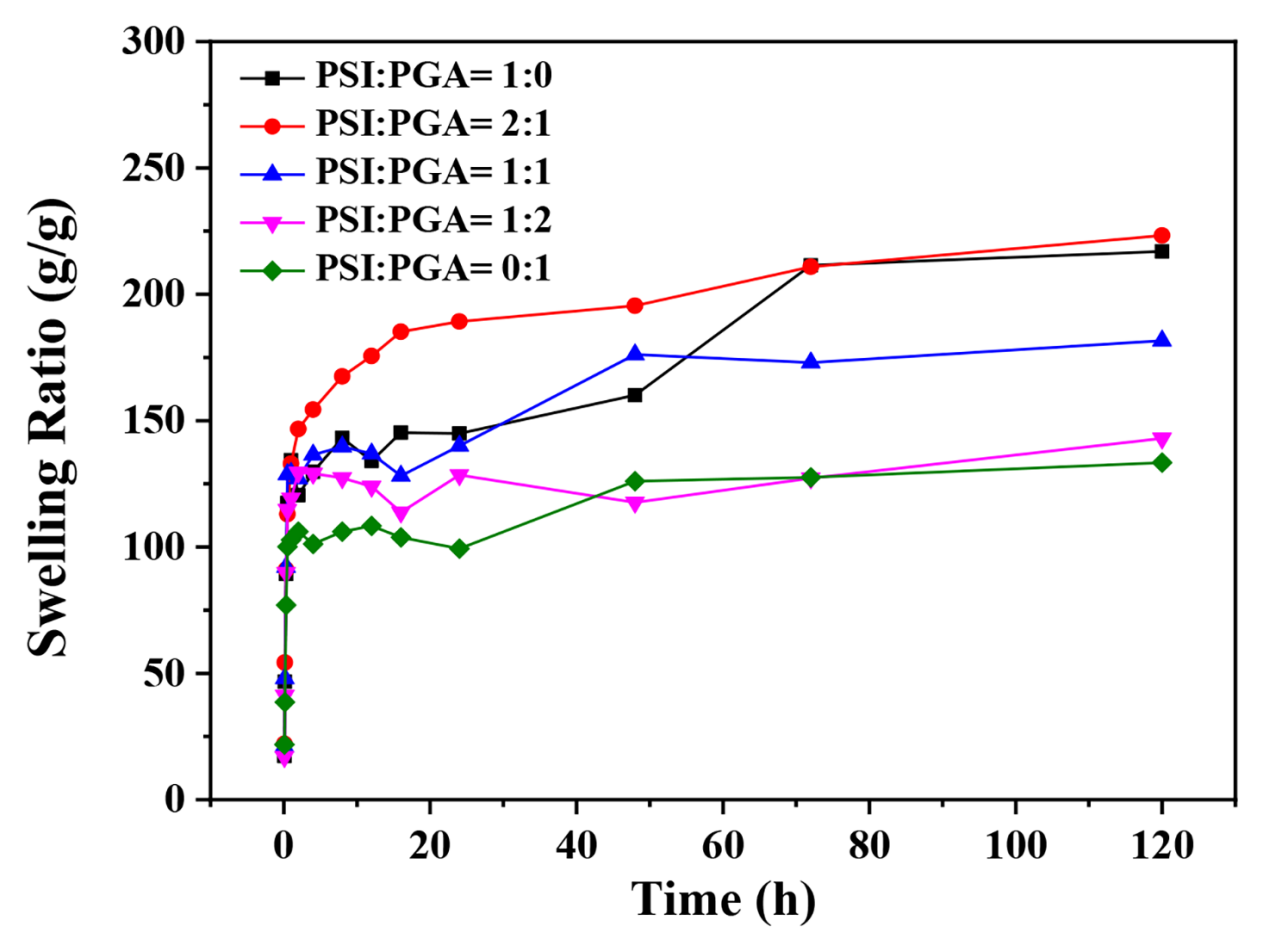
Swelling ratio of PASP and γ-PGA-co-PASP SAPSs obtained in deionized water

The time curves of the swelling rate of poly(amino acid) SAPs conducted in deionized water are plotted in Fig. 5. The entire backbone of poly(amino acid) SAPs was flexible, allowing the movement of the chains to facilitate the diffusion of water into the material, and the free carboxyl groups in the main chain also promoted polymer-water interactions, so poly(amino acid) SAPs showed excellent water absorption [28, 32]. Compared to PASP SAPs, the swelling ratio of γ-PGA-co-PASP SAPs also increased with time. When the solid-liquid ratio of PSI to γ-PGA fermentation liquid was 2:1, the γ-PGA-co-PASP SAPS had the highest water absorption. At 120 h, the maximum water absorption was 223 g/g, which was not different from that of pure polyaspartic acid SAPSs. The main reason for this was that the longer γ-PGA chain inserted into the PASP network could increase the pore size of the network and facilitate water absorption. With the increase of γ-PGA fermentation broth, the water absorption of γ-PGA-co-PASP SAPs decreased. A possible reason for this is that, although the inorganic salts in the fermentation broth of γ-PGA were removed by soaking and swelling, some residue remained. In addition, the bacterial residue contained in the γ-PGA fermentation broth may also have affected the swelling ability of the polymer.

### SEM observations

**Fig. 6:**
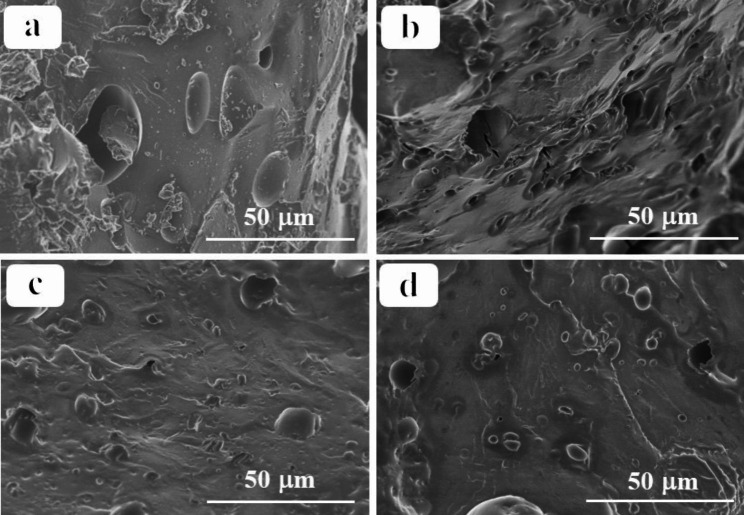
The surfaces of PASP SAPS (**a**) and γ-PGA-co-PASP SAPSs (**b**: PSI:PGA = 2:1; **c**: PSI:PGA = 1:1; **d**: PSI:PGA = 1:2).

Figure 6 shows the appearance and morphology of γ-PGA-co-PASP SAPs: the pure PASP SAPs demonstrate a smooth surface and a pore structure with a larger particle size (Fig. 6a), which ensures that PASP SAPs have excellent water absorption capacity. In addition, the surface of γ-PGA-co-PASP SAPs contained wrinkles and a cast-like shape with more uneven pores. As the amount of γ-PGA fermentation broth increased, the surface roughness increased, which might be due to the destruction of the regularity of the polymer surface structure by the residual inorganic salts and bacterial residues in the γ-PGA fermentation broth (Fig. 6b and c, and 6d). When the regularity of the polymer was destroyed to even a small extent, the water absorption points increased, and the density of the network structure decreased, which was conducive to the infiltration of water molecules. However, when the regularity was destroyed to a greater extent, the cations in the fermentation broth inhibited the infiltration of water molecules, and the low density of the network structure would lead to the ready loss of infiltrated water and a significant decrease in water absorption, which was consistent with the experimentally measured water absorption of γ-PGA-co-PASP SAPs [33].

### Thermal properties

**Fig. 7:**
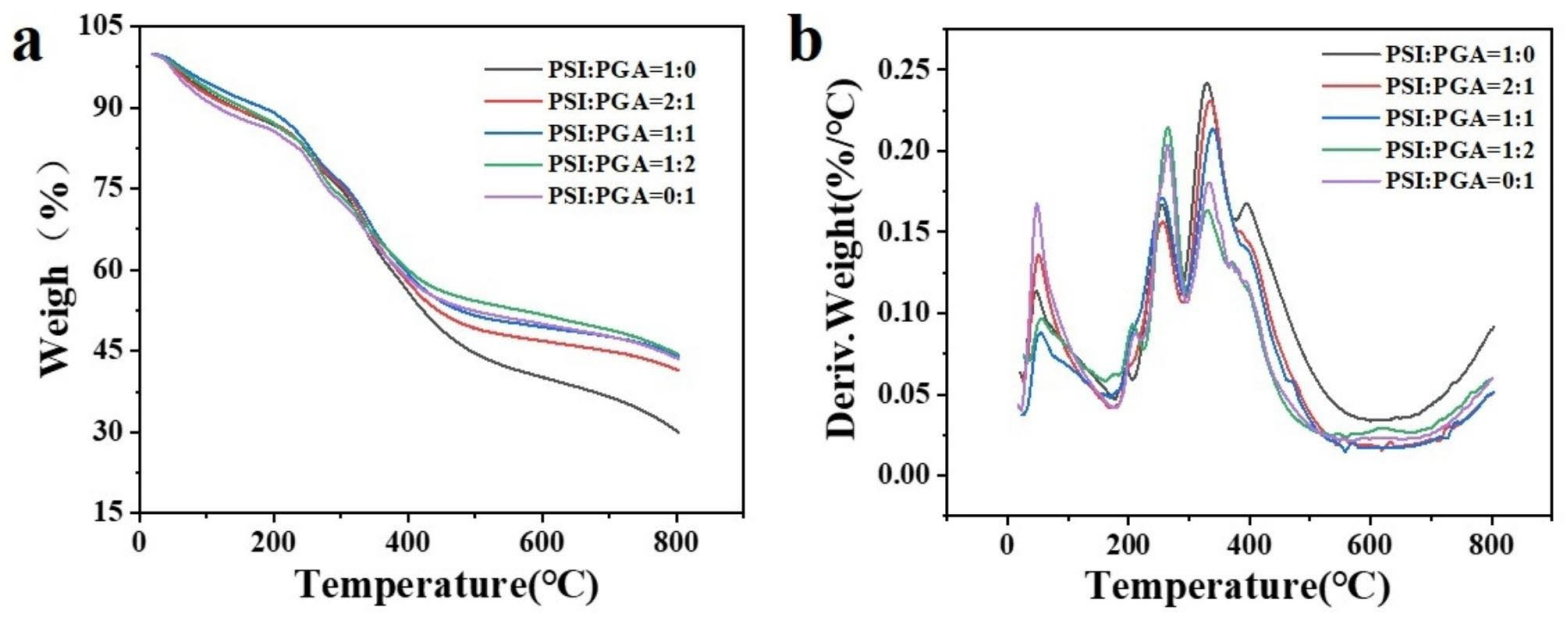
TGA (**a**) and DTG (**b**) curves of poly(amino acid) SAPSs

The changes of PASP SAPs before and after modification were evaluated by thermogravimetric analysis. As shown in Fig. 7, the trend in the thermogravimetric curves of PASP SAPs and γ-PGA-co-PASP SAPs is similar. The first stage was from 20 to 190 °C and showed an approximately 15% loss in weight, which could be attributed to the evaporation of adsorbed water and bound water; in the second stage, the weight loss began at 200 °C and continued to about 290 °C, and the weight lost from the sample was about 10%, which was mainly due to the dehydration of the polymer chain and the fracture of C-O-C bonds; the third stage follows, lasting until 560 °C. The weight loss of PASP SAPs was about 45%, while that of γ-PGA-co-PASP SAP was about 35%, which might be caused by the fracture of the main chain and the elimination of CO and CO_2_ [34]. As the temperature continued to rise, the residual organic matter continued to degrade. At 800 °C, the mass loss of PASP SAPs exceeded 70%, while the mass loss of γ-PGA-co-PASP SAPs remained less than 60% (Fig. 7a). γ-PGA has better heat resistance than PASP, and the heat resistance of the polyamino acid resin modified with γ-PGA is significantly higher than that of PASP SAPs. This is mainly due to the longer PGA molecular chain, which is conducive to the formation of a stronger and better thermal stability hydrogel skeleton. From Fig. 7b, it can be seen that in the first weight loss stage, PSI: PGA has the fastest weight loss rate at 0:1, mainly due to the higher liquid content in the γ-PGA fermentation broth. In the second stage, when the PSI: PGA ratio is 1:2, exhibits higher heat resistance, primarily due to the longer γ-PGA molecular chain and the sparser cross-linking network that is formed, which is more conducive to the fracture of large molecular chains. In the third stage, the PSI:PGA ratio of 2:1 shows the most significant weight loss, which may be due to the larger number of short-chain PASPs in the poly(amino) acid, thus making it easier for carbonaceous materials to undergo pyrolysis and oxidation and resulting in lower residual mass.

### Rheological properties

**Fig. 8:**
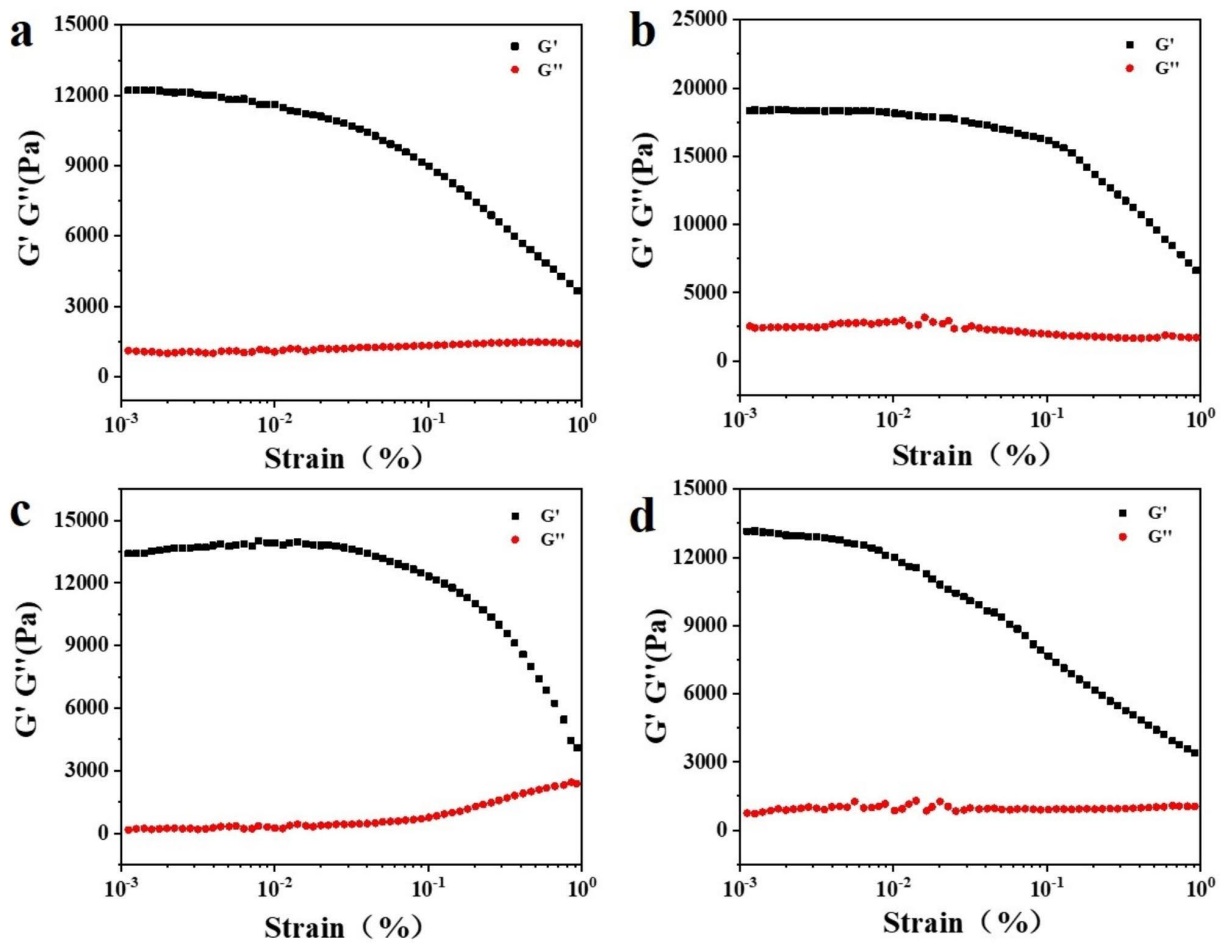
Strain scanning curve of γ-PGA-co-PASP SAPSs (**a**. PSI:PGA = 1:0; **b**. PSI:PGA = 2:1; **c**. PSI:PGA = 1:1, **d**. PSI:PGA = 1:2).

At room temperature, the oscillation frequency was 1 Hz, and the strain was between 10^− 3^% and 1%. The effects of the amount of PGA fermentation broth γ-PGA-co-PASP SAPs were investigated by strain scanning, and the variation in dynamic modulus with stress was determined (Fig. 8). After strain scanning, the SAPs showed a linear viscoelastic region, and the storage modulus (*G*´) of the resin was greater than the loss modulus (*G*´´), and the samples exhibited gel characteristics in this area [35, 36]. When the liquid-solid ratio of PSI to PGA was 2:1, the linear viscoelastic region increased, and the mechanical properties of the samples were optimal. When the strain was 0.1%, the strain-sweep curve dropped, and the sample structure was destroyed, so the strain of 0.1% was selected as that for subsequent tests.

**Fig. 9:**
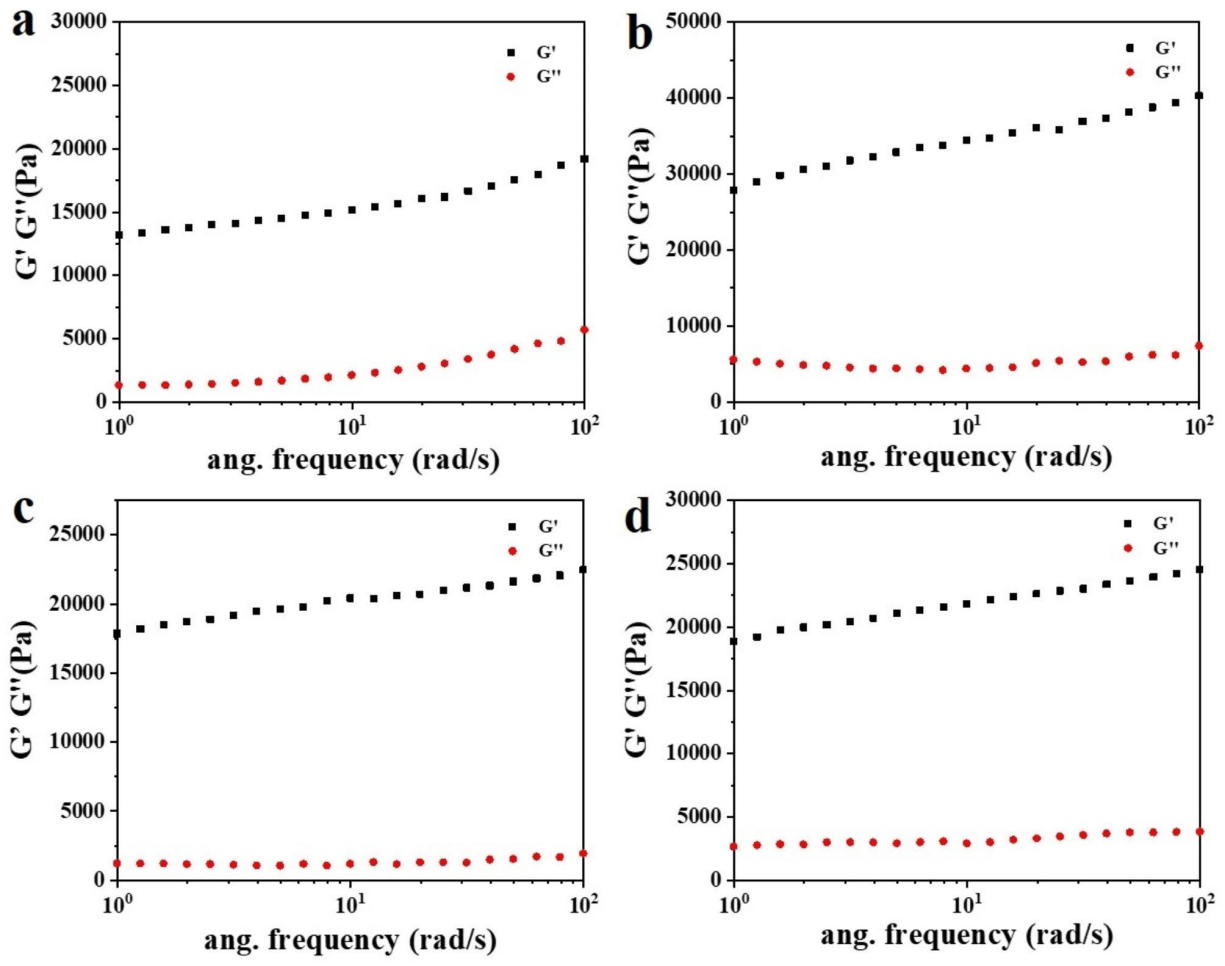
Frequency sweep curve of γ-PGA-co-PASP SAPSs (**a**. PSI:PGA = 1:0; **b**. PSI:PGA = 2:1; **c**. PSI:PGA = 1:1, **d**. PSI:PGA = 1:2).

Frequency sweeping was used to ascertain the rheological properties of elastomers under strain. At room temperature, the strain was 0.1%, the angular frequency was 0.01 ~ 100 rad/s, and the frequency sweep of SAPs was performed (Fig. 9): within the test range, the storage modulus (*G*´) of SAPs was much greater than the loss modulus (*G*´´), and both SAPs exhibited the characteristics of elastomers [37]. The values of *G*´ and *G*´´ of the modified PGA fermentation broth were higher than those of the unmodified SAPs, indicating that the modified PASP network structure has been further strengthened, giving it greater viscoelasticity. When the liquid-solid ratio of PSI: PGA was 2:1, the dynamic modulus of SAPs was the largest, which was mainly due to the excellent flexibility of the PGA molecular chain, enhanced chain entanglement, and increased dosage of PGA fermentation broth. Excessive metal cations would consume the carboxyl groups on PASP and PGA, which was not conducive to the formation of a stable network structure [38, 39].

## Conclusion

Polyglutamic acid was produced by using glutamic acid fermentation broth instead of pure glutamic acid, and then γ-PGA-co-PASP SAPs were prepared by using PSI as raw material, EGDGE as a crosslinking agent, and polyglutamic acid fermentation broth as a solvent. It was found that centrifugation can remove *C. glutamicum* from the glutamate fermentation broth. γ-PGA production decreased by about 5%. Solid substances in fermentation broth do not affect the synthesis of hydrogels but would affect the spatial structure of γ-PGA-co-PASP SAPs. When the PSI:PGA liquid-solid ratio was 2:1, PASP and PGA molecular chains were intertwined, and the water absorption was not different from that of pure PASP. SEM photos also showed that γ-PGA-co-PASP SAPs have a coarser surface and greater heat resistance and viscoelasticity. Using glutamic acid fermentation broth instead of glutamic acid powder for coupling fermentation can not only save the cost of separation and purification of glutamic acid but can also save part of the cost of glucose and inorganic salts. In addition, coupled fermentation can also avoid the waste of water resources in the fermentation process. γ-PGA-co-PASP SAPs have the advantages of low cost, a simple process, and an excellent water absorption effect, so it has good application potential.

## Data Availability

The datasets used and/or analyzed during the current study are available from the corresponding author upon reasonable request.

## Abbreviations

γ-PGA: γ-polyglutamic acid
PASP: polyaspartic acid
SAPs: superabsorbent polymers
γ-PGA-co-PASP: copolymer of γ-polyglutamic acid and polyaspartic acid
DCW: dry cell weight
PSI: polysuccinimide
*G*’: the storage modulus
*G*’’: the loss modulus

## References

[1] Zhang Z, Abidi N, Lucia L (2023). Smart superabsorbent alginate/carboxymethyl chitosan composite hydrogel beads as efficient biosorbents for methylene blue dye removal. J Mater Sci Technol.

[2] Hu XS, Wang CY, Yu HY (2023). Sodium tungstate as green cross-linker to improve water absorbency of superabsorbent polymer. J Appl Polym Sci.

[3] Ma X, Wen G (2020). Development history and synthesis of super-absorbent polymers: a review. J Polym Res.

[4] Abdallah AM, Mashaheet AM, Burkey KO (2021). Super absorbent polymers mitigate drought stress in corn (Zea mays L.) grown under rainfed conditions. Agr Water Manage.

[5] Chang L, Xu L, Liu Y, Qiu D (2020). Superabsorbent polymers used for agricultural water retention. Polym Test.

[6] Czarnecka E, Nowaczyk J (2020). Semi-natural superabsorbents based on starch-g-poly(acrylic acid): modification, synthesis and application. Polymers-Basel.

[7] Farzanian K, Vafaei B, Ghahremaninezhad A (2019). The behavior of superabsorbent polymers (SAPs) in cement mixtures with glass powders as supplementary cementitious materials. Materials.

[8] Capezza AJ, Malin L, Olsson RT, Newson WR, Hedenqvist MS, Eva J (2020). Carboxylated wheat gluten proteins: a green solution for production of sustainable superabsorbent materials. Biomacromolecules.

[9] Sang WT, Cui SZ, Wang XB, Liu B, Li XX, Sun KJ, Peng H, Ma GF. Preparation and properties of multifunctional polyaspartic acid/waste paper fiber-based superabsorbent composites. 2022; 10(5): 108405.

[10] Fan XY, Zhang R, Sui SM, Liu XR, Liu J, Shi CS, Zhao NQ, Zhong C, Hu WB (2023). Starch-based superabsorbent hydrogel with high electrolyte retention capability and synergistic interface engineering for long-lifespan flexible zinc-air batteries. Angew Chem Int Edit.

[11] Sand A, Vyas A (2020). Superabsorbent Polymer based on guar gum-graft-acrylamide: synthesis and characterization. J Polym Res.

[12] Mignon A, Belie ND, Dubruel P, Vlierberghe SV (2019). Superabsorbent polymers: a review on the characteristics and applications of synthetic, polysaccharide-based, semi-synthetic and ‘smart’ derivatives. Eur Polym J.

[13] Nakano T, Saito N, Minami H (2020). Preparation of cross-linked monodisperse poly(acrylic acid) particles by precipitation polymerization. Langmuir.

[14] Djafari SR, Vatankhah E (2020). Environmentally friendly superabsorbent fibers based on electrospun cellulose nanofibers extracted from wheat straw. Carbohyd Polym.

[15] Sorokin A, Lavlinskaya M (2021). Synthesis of the superabsorbent enriched in chitosan derivatives with excellent water absorption properties. Polym Bull.

[16] Ravishankar K, Dhamodharan R (2020). Advances in chitosan-based hydrogels: evolution from covalently crosslinked systems to ionotropically crosslinked superabsorbents. React Funct Polym.

[17] Wang XH, Li K, Zhang XY, Gao T, Zhang L, Shen YL, Yang L. Performance of chitosan/γ-polyglutamic acid/curcumin edible coating and application in fresh beef preservation. Ciencia Tecnol Alime. 2023;43. 10.1590/fst.103222.

[18] Kim HC, Kim E, Hong BM, Park SA, Park WH (2021). Photocrosslinked poly(γ-glutamic acid) hydrogel for 3D bioprinting. React Funct Polym.

[19] Li Z, He G, Hua J, Wu M, Guo W, Gong J, Zhang J, Qiao C (2017). Preparation of γ-PGA hydrogels and swelling behaviors in salt solutions with different ionic valence numbers. RSC Adv.

[20] Zhang M, Wang W, Wu F, Zheng T, Sun Y (2020). Biodegradable poly(γ-glutamic acid)@glucose oxidase@carbon dot nanoparticles for simultaneous multimodal imaging and synergetic cancer therapy. Biomaterials.

[21] Su Z, Han C, Liu E, Zhang F, Meng X (2021). Formation, characterization and application of arginine-modified chitosan/γ-poly glutamic acid nanoparticles as carrier for curcumin. Int J Biol Macromol.

[22] Fang J, Liu Y, Huan CC, Xu L, Yan Z (2020). Comparison of poly-γ-glutamic acid production between sterilized and non-sterilized solid-state fermentation using agricultural waste as substrates. J Clean Prod.

[23] Li TZ, Tan ZJ, Tang ZJ, Liu P, Liu HF, Zhu LL, Ma YH (2022). One-pot chemoenzymatic synthesis of glycolic acid from formaldehyde. Green Chem.

[24] Wei J, Zhao JB, Cai D, Ren WQ, Cao H, Tan TW (2020). Synthesis of micro/meso porous carbon for ultrahigh hydrogen adsorption using cross-linked polyaspartic acid. Front Chem Sci Eng.

[25] Zhao JB, Liang XX, Cao H, Tan TW (2020). Preparation of injectable hydrogel with near-infrared light response and photo-controlled drug release. Bioresour Bioprocess.

[26] De Grave L, Tenorio JR, Snoeck D, Vynnytska S, De Belie N, Bernaerts KV, Van Vlierberghe S (2022). Poly(aspartic acid) superabsorbent polymers as biobased and biodegradable additives for self-sealing of cementitious mortar. J Sustain Cem-Based.

[27] Zhao JB, Wei J, Cai D, Cao H, Tan TW (2020). Polyaspartic acid-derived micro-/mesoporous carbon for Ultrahigh H_2_ and CH_4_ adsorption. ACS Omega.

[28] Zhao JB, Wei J, Zhang YW, Tan TW, Nie KL, Cao H (2019). Development of a polyaspartic acid hydrogel fabricated using pickering high internal phase emulsions as templates for controlled release of Drugs. J Biobased Mater Bio.

[29] Prell C, Busche T, Rückert C, Nolte L, Wendisch VF (2021). Adaptive laboratory evolution accelerated glutarate production by corynebacterium glutamicum. Microb Cell Fact.

[30] Qiu Y, Aierzhati A, Cheng J, Guo H, Zhang Y (2019). Biocrude production through maillard reaction between leucine and glucose during the hydrothermal liquefaction. Energy Fuels.

[31] Tsuge Y, Matsuzawa H (2021). Recent progress in production of amino acid-derived chemicals using Corynebacterium glutamicum. World J Microb Biot.

[32] Kian LK, Jawaid M, Alamery S, Vaseashta A (2021). Fabrication and characterization of novel poly(D-lactic acid) nanocomposite membrane for water filtration purpose. Nanomaterials.

[33] Zhang Y, Li C, Jia R, Gao R, Zhao Y, Ji Q, Cai J, Li Q, Wang Y (2021). PEG-poly(amino acid)s/EpCAM aptamer multifunctional nanoparticles arrest the growth and Metastasis of Colorectal cancer. Biomater Sci.

[34] Xu JC, Kim KO, Yoon KJ (2022). Effect of cross-linker length on the absorption characteristics of the sodium salt of cross-linked polyaspartic acid. Polymers.

[35] Zheng QY, Xie B, Xu ZL, Wu H (2023). A systematic printability study of direct ink writing towards high-resolution rapid manufacturing. Int J Extreme Manuf.

[36] Vu T, Reynolds G, Hutton H, Kasting GB, Koenig P (2021). Rheology control using nonionic cosurfactants and pH titration in an amino acid-derived surfactant composition. Langmuir.

[37] Park SB, Sung MH, Uyama H, Han DK (2021). Poly(glutamic acid): production, composites, and medical applications of the next-generation biopolymer. Prog Polym Sci.

[38] Li GF, Shi Z, Zong HJ, Zhang KX, Yan SF, Yin JB (2023). Injectable, self-healing poly(amino acid)-hydrogel based on phenylboronate ester bond for osteochondral tissue engineering. Biomed Mater.

[39] Lin ZQ, Ding JF, Chen XS, He CL. pH- and temperature-responsive hydrogels based on tertiary amine-modified polypeptides for stimuli-responsive drug delivery. Chem-Asian J. 2023;18(8). 10.1002/asia.202300021. 10.1002/asia.202300021 36856525

